# Physical Variables Underlying Tactile Stickiness During Fingerpad Detachment

**DOI:** 10.3389/fnins.2020.00235

**Published:** 2020-04-15

**Authors:** Saekwang Nam, Yasemin Vardar, David Gueorguiev, Katherine J. Kuchenbecker

**Affiliations:** Haptic Intelligence Department, Max Planck Institute for Intelligent Systems, Stuttgart, Germany

**Keywords:** finger, mechanics, stickiness, perception, pressing time, separation time, impulse, pull-off

## Abstract

One may notice a relatively wide range of tactile sensations even when touching the same hard, flat surface in similar ways. Little is known about the reasons for this variability, so we decided to investigate how the perceptual intensity of light stickiness relates to the physical interaction between the skin and the surface. We conducted a psychophysical experiment in which nine participants actively pressed their finger on a flat glass plate with a normal force close to 1.5 N and detached it after a few seconds. A custom-designed apparatus recorded the contact force vector and the finger contact area during each interaction as well as pre- and post-trial finger moisture. After detaching their finger, participants judged the stickiness of the glass using a nine-point scale. We explored how sixteen physical variables derived from the recorded data correlate with each other and with the stickiness judgments of each participant. These analyses indicate that stickiness perception mainly depends on the pre-detachment pressing duration, the time taken for the finger to detach, and the impulse in the normal direction after the normal force changes sign; finger-surface adhesion seems to build with pressing time, causing a larger normal impulse during detachment and thus a more intense stickiness sensation. We additionally found a strong between-subjects correlation between maximum real contact area and peak pull-off force, as well as between finger moisture and impulse.

## 1. Introduction

Tactile interactions occur so often that humans rarely recognize their importance. Our daily tactile interactions start in the morning when we reach to turn off our alarm clock and continue during the day with physical contacts mediating our every action in the real world. They also give us a window into the digital: on average, a person touches his or her mobile phone 2,617 times per day (Winnick, 2016), though the frequency of these actions often remains unnoticed. However, when one pays close attention, one may notice a relatively wide range of tactile sensations even when the finger is touching the same object in similar ways. For example, the surface of a screen or a cup can sometimes feel sticky and sometimes not. During dynamic touch, the stickiness of a surface is commonly related to the finger-surface friction and can depend on the touched material (Bensmaïa and Hollins, 2005), the characteristics of the fingerpad (Dinç et al., 1991; Cornuault et al., 2015), and exploratory parameters, such as the sliding velocity and the contact force (Tang et al., 2015a; Ben Messaoud et al., 2016). However, the stickiness of a material can also be felt during static touch, probably through adhesion and micro-stretching of the skin (Bergmann Tiest, 2010). Variations in the mechanical properties of the finger are also known to significantly impact stickiness perception (Demartine and Cussler, 1975). Therefore, we believe the perceptual intensity of stickiness can be understood by investigating the physical interaction between the fingerpad and the surface.

The adhesion and detachment dynamics of a finger on a surface are mediated by the physiology of the finger and the physical bonds that are created. Therefore, research in physics and materials science can give us hints about the physical variables that affect the perception of stickiness. The American Society for Testing and Materials defined sticky or tacky materials as those that need additional force to separate from another item immediately after the creation of contact (Gay and Leibler, 1999). Many adhesion-based interpretations have also been proposed to explain stickiness (Gay, 2002; Pastewka and Robbins, 2014). Some adhesion theories, such as Derjaguin-Muller-Toporov (DMT) and Johnson-Kendall-Roberts (JKR), use fundamental principles to derive the force required to separate an elastic body from a hard body. These theories provide ways to calculate the pull-off force based on the contact area, pressing force, and material properties of both objects (Barthel, 2008); their predictions were later supported by experimental measurements (Dorogin et al., 2017, 2018). Johnson also extended these theories to the case of viscoelastic materials (Johnson, 1999). However, these approaches are not perfectly applicable to fingertip interactions because the finger's physical contact conditions change over time. For example, researchers recently found that moisture secretion from sweat glands softens the stratum corneum, the outermost layer of the fingerpad (Dzidek et al., 2017).

When it comes to the perception of stickiness, little is known about its perceptual dimensions, which makes it difficult to understand the underlying mechanisms. Zigler was one of the first to study stickiness perception using psychophysical experiments, where participants pressed their fingertips on sticky materials, such as liquid glue, prunes, molasses, and jelly (Zigler, 1923), and described their experiences. This experiment revealed that participants distinguished stickiness by expressing “pull” in the case of strong stickiness and “breakaway” for light, superficial stickiness. In later psychophysical experiments, Bensmaïa and Hollins suggested that the human perception of stickiness/slipperiness is mediated by intensive representations of the tactile signals, which are possibly encoded by the Pacinian tactile afferents (Bensmaïa and Hollins, 2005). Recently, Mith et al. (2008) conducted experiments where participants rated the tackiness intensity of a set of silicone elastomer sheets. Mith et al. then measured the indentation force and depth when a probe pressed into the same samples, and they correlated the perceptual intensity judgments with the adhesion parameters, finding that human tackiness intensity is highly correlated with the full distance over which the probe separates from the elastomer. Even more recent studies have focused on the neural correlates of stickiness by observing neural activity in the human brain using functional magnetic resonance imaging (fMRI) (Yeon et al., 2017). They discovered that the activated brain areas differ depending on the intensity of stickiness. In particular, two psychophysical experiments (method of constant stimuli, magnitude estimation) led them to divide sticky stimuli into three groups. These groups were used to conduct a contrast fMRI analysis that found that there are comparably more active brain areas during interactions with stickier surfaces. Kim et al. conducted multivoxel pattern analysis on the contrast analysis data and showed distinct neural activity patterns depending on the stickiness intensity (Kim et al., 2017).

In general, previous experiments on stickiness perception used highly sticky materials so that human subjects could perceive vivid signals. However, as far as we are aware, earlier studies did not explore the more subtle effect of how the perceived stickiness of a lightly sticky surface changes due to physical interaction conditions. To fill this gap, we investigate the physical variables that affect the perception of stickiness in a particular touch interaction (i.e., the finger detaches from a flat, hard glass surface). To understand the connection between perception and mechanics, we conduct a psychophysical experiment in tandem with physical measurements using a custom apparatus designed for active touch. Based on past research showing the factors that mainly affect adhesion between rigid and viscoelastic materials (Johnson, 1999), we define sixteen physical variables and explore correlations between these variables and the stickiness judgments of the participants. By analyzing the correlations, we show that stickiness perception mainly depends on the pre-detachment pressing time, the time taken for the finger to detach, and the impulse in the pressing (normal) direction during the finger detachment after the normal force changes sign.

This paper is organized as follows: in section 2, we describe our experimental apparatus, define sixteen physical variables of interest, and outline the methods for our human participant experiment. Section 3 presents the physical variables, the perceptual stickiness ratings, and how they both vary across trials and participants. Section 4 discusses the results, particularly which physical variables the participants considered when rating stickiness in this experiment, and section 5 concludes the paper.

## 2. Materials and Methods

In this section, we first introduce a custom-made apparatus for the measurement of three-degree-of-freedom (3-DoF) force and contact fingerprint images. Then, the statistics of the participants and the experimental procedures are described. Lastly, we explain how we process the raw data measured during each trial to derive the sixteen physical variables that we expect may relate to the perception of stickiness.

### 2.1. Apparatus

**Figure 1:**
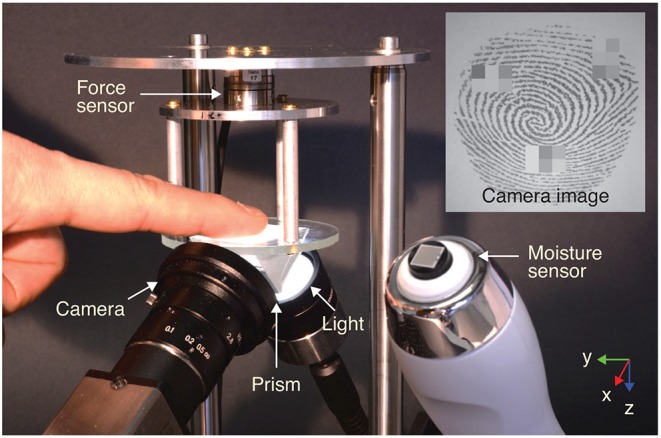
Apparatus for measuring the moisture content, contact force vector, and real contact area of a finger actively pressing on a glass plate. Note that three small rectangular regions were pixelated in the fingerprint image before publication to conceal the identity of this participant.

We designed an experimental apparatus that can measure contact forces and finger contact area over time to test how these quantities are related to human stickiness perception (see Figure 1). We also measured the moisture of the participant's fingerpad, as moisture tends to change physical interactions between the skin and a surface (Tomlinson et al., 2011; Derler et al., 2015; Dzidek et al., 2017; Gueorguiev et al., 2017). Moreover, we took the comfort of the participants into account by not attaching any fixtures to the finger and by making the finger-glass interaction direction downward.

A strain-based force sensor (Nano17 Titanium SI-32-0.2, ATI Inc.) was mounted above a glass surface to measure the contact force vector with a resolution of 1/171 N in all directions. The force data were collected by a data acquisition board (PCIe 6323, NI Inc.) with a sampling rate of 500 Hz. The non-coated soda lime silicate glass plate (Optifloat™ Clear, Pilkington Deutschland AG) had a thickness of 3 mm and a roughness <10 nm (Gläser, 1999). An optical monochrome image sensor (DCC2340M, Thorlabs Inc.) with a lens (MVL5M23, Thorlabs Inc.) was installed below the glass plate to measure the contact area of the fingerpad. The recording frame rate of the camera was set to 10 frames per second. The aperture size of the lens was minimized (*f*/16) to maximize the depth of focus, providing a larger focused area.

The light intensity contrast between the contact and non-contact fingerprint areas was emphasized by applying prism-based frustrated total internal reflection (FTIR) (Levesque and Hayward, 2003; Bochereau et al., 2017). In this configuration, most of the light propagating through the prism is reflected at the top surface of the glass plate, but the light is scattered where contact occurs between the fingerprint and the glass surface, resulting in low light intensity (dark) at the contacted points in each image (see the inset image in Figure 1) (Bochereau et al., 2017). The prism was glued beneath the glass substrate with cured polydimethylsiloxane (PDMS). For that, a mixture of a pre-polymer and a cross-linker of Dow Corning® Sylgard 184 with a ratio of 10:1 was degassed and cured in an oven for 1 h at 90°C. The contact surface was illuminated from below by a light source (KL 2500 LED, SCHOTT AG) shining through a light diffuser attached to the prism surface, yielding an evenly bright background. The fingerpad moisture was separately measured with a capacitive-type moisture sensor (Corneometer® CM 825 w, Courage + Khazaka electronic GmbH) installed next to the contact glass; this sensor measures the moisture value of the outer-most layer of the fingerpad in arbitrary units (a.u.) between 0 and 130 (Constantin et al., 2014). Two custom-made LabVIEW programs (Version 2018 18.0f1) simultaneously collected the real-time data measured by the force sensor and the camera. The software was operated on 64-bit Windows 10 Enterprise 2016 LTSB installed on a computer with an Intel® Core™ i7-7700 CPU and 32 GB RAM.

### 2.2. Participants

Ten people (three women, seven men) with a mean age of 29 years (standard deviation, SD: 6.4 years) participated in the experiments. None of them had current or past sensory or sensory-motor disabilities. All participants were right handed. The participants provided informed consent and received no compensation.

### 2.3. Experimental Procedure

Each participant took a seat after washing and drying their hands. First, participants familiarized themselves with the target interaction using the index finger of their dominant hand to touch a glass plate taped to the table. These interactions provided an opportunity to investigate the stickiness of the glass using their choice of exploratory procedures. It was the same material as the glass plate in the apparatus so that the participant could experience levels of stickiness similar to those of the experiment.

**Figure 2:**
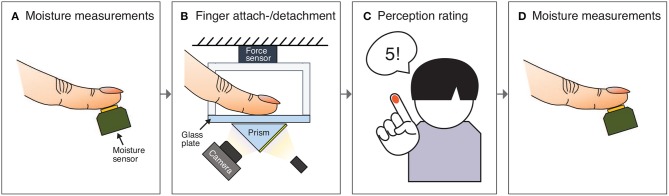
The chronological procedure of one trial of the experiment. After **(A)** three measurements of the fingerpad's moisture, **(B)** the participant places his or her finger on a clean glass plate and reaches a normal force of 1.5 N. After a preprogrammed pressing duration (0, 1.5, or 3 s), a visual cue appears on the screen prompting the participant to detach the finger. **(C)** The participant detaches his or her finger and judges the stickiness of the glass using a nine-point scale. **(D)** The participant's fingerpad moisture level is measured three more times.

Once the participant was familiar with the stickiness of the glass plate, he or she started the experimental procedure. The participant's dominant index fingerpad was wiped with isopropyl alcohol once at the start of the experiment to clean it, and the glass plate of the apparatus was cleaned before every trial. Then, the moisture level of the fingerpad was measured three times in quick succession (Figure 2A). After that, the participant placed his or her finger at the center of the glass plate of the apparatus while watching a visual indicator of normal force (Figure 2B). When they reached 1.5 N, which is considered a light pressing force in active touch (Papetti et al., 2017), the computer pseudo-randomly selected an additional pressing time of 0.0, 1.5, or 3.0 s. These three pressing times were chosen because physical contact on the fingerpad is strongly affected by sweat secreted in the first 10 s after initial contact (Pasumarty et al., 2011). After the additional pressing time, a visible cue appeared on the computer screen prompting the participant to detach his or her finger. After detaching, the participant verbally gave a stickiness rating ranging from 1 (not at all sticky) to 9 (highly sticky) (Figure 2C). Lastly, the participant's finger moisture level was again measured three times (Figure 2D). Each participant repeated this same procedure for 42 trials (14 trials × 3 different pressing durations). The average temperature in the laboratory was 21.7°C (SD: 1.1°C), and the average humidity was 50.3% (SD: 3.4%). The total duration of the experiment was about 1 h per participant.

### 2.4. Data Processing

The raw data collected from the force and image sensors were processed to compute the physical variables that we investigate in this study. First, time-stacked fingerprint image data underwent several processing steps for contact area extraction, and the obtained contact area was then synchronized with the recording of the finger-surface contact forces.

#### 2.4.1. Calculating the Real Contact Area

**Figure 3:**
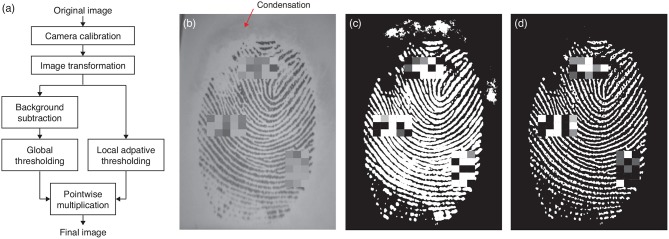
**(a)** Procedure for calculating the real finger contact area from a raw image. The camera calibration step corrects the lens distortion. Next, image transformation rectifies the slanted finger image. Two different threshold methods applied in parallel appropriately distinguish the finger contact area from the non-contact area without being confused by **(b)** condensed moisture around the finger. In such cases, taking **(c)** the global threshold identifies condensation as part of the contact finger area, but applying a local adaptive threshold removes the condensation area to produce **(d)** the final image. Note that three small rectangular regions were pixelated in each fingerprint image before publication to conceal the identity of this participant.

Real contact area is one of the main variables that might affect stickiness perception. To compute real contact area, we applied the series of image processing steps shown in Figure 3.

An image recorded by the camera cannot be directly used to calculate the contact area because it is distorted by both the round shape of the lens and the viewing angle between the camera and the glass plate. The radial image distortion due to the lens was flattened using camera calibration. This process made use of the intrinsic camera parameters computed from several images of a printed checkerboard taken from different angles (MATLAB Camera Calibration Toolbox, 2018). Next, the flattened image was transformed to estimate the image that the camera would see if it was positioned perpendicular to the glass plate. For that, we first captured a 15 × 15 mm image of a square rubber piece placed on the glass, and we then calculated the transformation matrix that converts the viewed shape to a square using the projective transformation method (Goshtasby, 1986). We used this same image to calculate the area per pixel (0.000986 mm^2^/pixel) by dividing the actual area of the square by the number of pixels it occupied in the transformed image.

The real finger contact area is usually calculated using global thresholding. In this method, the contact area is found by first subtracting what is seen before finger contact and then binarizing the image with a thresholding value. However, a highly moist finger causes the condensation of tiny liquid droplets around the finger (see Figure 3b), which look like vague clouds and were also detected as contact points by global thresholding (Figure 3c). We solved this issue by local adaptive thresholding (Figure 3d) (Davies, 2012), which calculates different threshold values for different regions of the image. We used the intersection of the two logical (negative/positive) images based on each thresholding method to obtain a binarized image that reflects real contact. Finally, we calculated real fingerpad contact area by multiplying the number of pixels considered as contact by the area per pixel.

#### 2.4.2. Extracting Parameters From the Force and Contact Area Data

**Figure 4:**
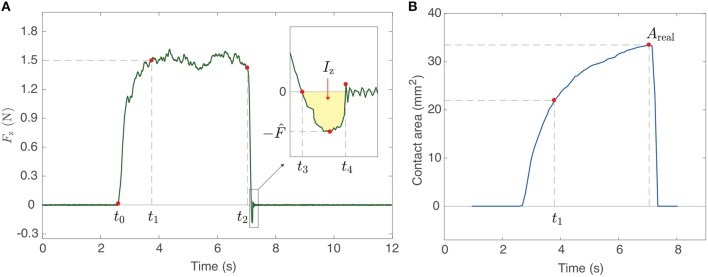
**(A)** The measured force in the z-direction and **(B)** the real contact area as a function of time from a sample trial, including parameter definitions.

We extracted key parameters from the measured force and contact area data (see red dots in Figure 4) after synchronizing the image and contact force data recordings. In order to extract quantitative measurements that relate to the physical variables under scrutiny, we decomposed the dynamics of pressing and detaching the finger into several qualitatively different time intervals. *t*_0_ is defined as the time the finger starts to contact the plate. The time the normal force reaches 1.5 N is marked as *t*_1_. *t*_2_ is the time the pressing finger starts to detach by reducing its normal force. The finger pulls off the stationary glass plate and usually generates negative force values for a short duration. This detachment phase occurs between *t*_3_ and *t*_4_ (inset in Figure 4A), where *t*_3_ is the time when the force is closest to 0 N during the detachment, and *t*_4_ is the first time when the finger is completely detached. We define the peak pull-off force as the minimum contact force in the z-direction ($\hat{F}$). The maximum real contact area before detachment is defined as *A*_real_.

#### 2.4.3. Physical Variables Investigated for Stickiness Perception

Based on the parameters extracted from the measured raw data, we defined sixteen physical variables to be investigated in correlation with stickiness perception. These variables were selected based on contact-adhesion theories and related experiments found in the literature, as noted.

1. **The finger holding duration while the pressing force is kept around 1.5 N (*t*_hold_ = *t*_2_ − *t*_1_)**
  *t*_hold_ is the duration between the instant that the force in the z-direction reaches 1.5 N (*t*_1_) and the time that the pressing force starts to decrease (*t*_2_). This holding time leads to changes in physical phenomena, such as sweat secretion, which is known to affect the softness of the fingerpad (Dzidek et al., 2017), and occlusion, which reduces water lost to the atmosphere (Zhai and Maibach, 2001; Pasumarty et al., 2011).
2. **Detaching duration of the finger after force changes sign (*t*_pull_ = *t*_4_ − *t*_3_)**
  Fast adapting (FA) mechanoreceptors are known to respond strongly when the applied mechanical stimulus changes (Vallbo and Johansson, 1984; Caldwell et al., 1999). Therefore, we thought looking at the time it takes the finger to pull off the glass plate is particularly important. This value is obtained by subtracting *t*_3_ from *t*_4_.
3. **Detachment rate (Ḟ)**
  Researchers previously showed that a glass ball's peak pull-off force from a polyurethane surface depends on its detachment speed (Barquins and Maugis, 1981). Because the materials are similar, we anticipate a similar result in our study. Since our apparatus cannot measure finger motion in the normal direction, our analysis uses the detachment rate [Ḟ (N/s)] as a similar variable. It shows how quickly the measured force decreases during finger detachment (from *t*_2_ to *t*_3_). To derive this value, we fit a linear function to the force data between the two time points, and we use the absolute value of the derivative of the function as the detachment rate.
4. **Detachment rate at *t*_3_ (Ḟ_*t*_3__)**
  Although separation of a fingerpad from the glass plate happens very quickly, the instantaneous detachment rate often changes across this time span. Focused more at the moment of the separation (*t*_3_), we define another variable regarding the detachment rate. It is calculated in the same way as Ḟ, but we consider the force rendered in the last 0.01 s before *t*_3_.
5. **Root mean square of force in the x-direction during the pull-off (*F*_x,rms_)**
  As shown in Figure 1, the x-axis points from the apparatus toward the participant's hand. Thus, the measured force in this direction is expected to come from friction between the finger and the glass. As this frictional force can affect stickiness perception (Yeon et al., 2017), we define a representative force value by taking the root mean square (RMS) of the x-force while the finger is experiencing a negative normal force (from *t*_3_ to *t*_4_). Therefore, if there are *n* values of *F*_x_ between *t*_3_ and *t*_4_, the variable is defined as $F_{\text{x},\text{rms}}=\sqrt{\frac{1}{n}\left({{F_{\text{x}}}_{1}}^{2}+{{F_{\text{x}}}_{2}}^{2}+\ldots +{{F_{\text{x}}}_{n}}^{2}\right)}$.
6. **RMS of force in the y-direction during the pull-off (*F*_y,rms_)**
  For the same reason mentioned above, we are also interested in the RMS of the interaction force in the y-direction. This value shows the strength of the frictional force a finger exerts to its left and right between *t*_3_ and *t*_4_. Therefore, $F_{\text{y},\text{rms}}=\sqrt{\frac{1}{n}\left({{F_{\text{y}}}_{1}}^{2}+{{F_{\text{y}}}_{2}}^{2}+\ldots +{{F_{\text{y}}}_{n}}^{2}\right)}$.
7. **RMS of force in the z-direction during the pull-off (*F*_z,rms_)**
  The force in the z-direction shows the interaction between the finger and the plate in the normal direction. During pull-off, it highlights the additional force needed to separate the finger from the glass plate (Pastewka and Robbins, 2014). Like *F*_x,rms_ and *F*_y,rms_, the definition for the z-direction is $F_{\text{z},\text{rms}}=\sqrt{\frac{1}{n}\left({{F_{\text{z}}}_{1}}^{2}+{{F_{\text{z}}}_{2}}^{2}+\ldots +{{F_{\text{z}}}_{n}}^{2}\right)}$.
8. **RMS of force during the pull-off (*F*_rms_)**
  People could perceive stickiness without considering the direction in which the force occurs (i.e., based on the magnitude of the force vector), particularly in the case when the interaction time is very short, such as finger detachment. Thus, comparing the RMS force in each direction (*F*_x,rms_, *F*_y,rms_, *F*_z,rms_) with the RMS of the force vector's magnitude (*F*_rms_) can tell us whether a particular force direction (such as frictional or normal) is crucial for people to feel stickiness. The value is calculated as $F_{\text{rms}}=\sqrt{\frac{1}{n}\left({F_{1}}^{2}+{F_{2}}^{2}+\ldots +{F_{n}}^{2}\right)}$, where each *F* is force vector magnitude.
9. **Impulse in the x-direction during the pull-off (*I*_x_)**
  The RMS force calculations average over time and therefore disregard the wide range of pull-off times (*t*_pull_). We define the pull-off impulse as the integral of force over time to reflect both the measured time and force; such a calculation is common in mechanics, but we have not found any prior use of impulse in stickiness research. Here, we consider only the x-direction, and we take the absolute value of the force because we believe forces in both directions may cause a sensation of stickiness. Therefore, *I*_x_ is calculated as $\int_{t_{3}}^{t_{4}}|F_{\text{x}}\left(t\right)|dt$.
10. **Impulse in the y-direction during the pull-off (*I*_y_)**
  Impulse in the other frictional direction is also considered as a physical variable. Therefore, *I*_y_ is $\int_{t_{3}}^{t_{4}}|F_{\text{y}}\left(t\right)|dt$.
11. **Impulse in the z-direction during the pull-off (*I*_z_)**
  The normal direction of impulse during the finger's detachment shows the total adhesive force applied to the finger. As the force in the z-direction during the pull-off is always negative, we flipped its sign so that the magnitude of the z-impulse is intuitively matched to the perception of stickiness. One example of the value is shown as the area shaded in yellow in the inset of Figure 4A. *I*_z_ is $\int_{t_{3}}^{t_{4}}-F_{\text{z}}\left(t\right)dt$.
12. **Total impulse during the pull-off (I)**
  For the same reason that we set *F*_rms_ as one of the physical variables, we also define the impulse of the force vector magnitude to combine all three directions. Mathematically, this is $\int_{t_{3}}^{t_{4}}\sqrt{{F_{\text{x}}\left(t\right)}^{2}+{F_{\text{y}}\left(t\right)}^{2}+{F_{\text{z}}\left(t\right)}^{2}}dt$.
13. **Maximum real contact area (*A*_real_)**
  Under the same conditions for the pressing force, time, and material properties of a viscoelastic finger, differences in the contact area are known to produce meaningful distinctions in contact adhesion (Barthel, 2008). The maximum value can be found from the measured contact area as a function of time (the red dot indicating *A*_real_ in Figure 4B).
14. **Peak pull-off force ($\hat{F}$)**
  As adhesion theories have evolved based on the study of adhesive force between two contacting objects (Barthel, 2008; Dorogin et al., 2017), we thought pull-off force could be highly related with stickiness. As shown in the inset of Figure 4A, *F*_z_ is negative when a finger is pulling off the glass plate. The most negative force in the inset is considered as the peak pull-off force ($\hat{F}$). Here, we made $\hat{F}$ positive by flipping the sign of *F*_z_ so that larger values are intuitively connected to the magnitude of stickiness.
15. **Mean moisture value ($\bar{M}$)**
  Sweat secretion from the pores located along the fingerprint ridges can contribute to better grip by strengthening the coalescence process between the fingerpad and the contact surface (Dzidek et al., 2017). Thus, moisture-related variables should be taken into consideration in our analyses. We measured the moisture value six times in each trial (three times before, three times after) using the moisture sensor. The mean moisture value comes from taking the average of the six measured values.
16. **Change in moisture after finger detachment (Δ*M*)**
  The change in the amount of sweat on the skin might also influence the perception of stickiness. Here, Δ*M* of a given trial is the difference between the average moisture value measured after the trial and the average value before the trial, with positive variable values indicating an increase in moisture.

**Stickiness ratings** (Rating). After each trial, the participant rated the stickiness they experienced using a nine-point scale (1–9), with larger values meaning a stickier interaction.

#### 2.4.4. Data Analysis

We applied Spearman's rank-order correlation to discover which of the sixteen calculated physical variables are perceptually related to stickiness and how these variables are correlated with each other. This method elucidates how closely the ranked values of one physical variable match the rank order of another variable. Given pairs of a measured value and a rating, where the measured values are sorted in ascending order, the correlation of the pairs is high (ρ≈1) if the order of the ratings is also close to ascending order. This coefficient (ρ) is 0 when there is no order similarity at all between the two lists of values, and it is −1 when they are anti-correlated. We visualize the correlations between all possible pairs of a participant's sixteen variables and ratings as a heatmap. An additional heatmap presents the correlations between the median values of the physical variables across participants.

The reliability of the correlation analysis presupposes that all physical conditions, such as the finger's temperature and material properties, do not change significantly across trials. Because the isopropyl alcohol used to clean the finger at the start of the experiment might affect the physical status of the finger, and also because fingers quickly adapt to the interaction condition by changing the speed of sweat secretion (Johansson and Cole, 1994), we discarded the data from the first six trials of each participant, leaving about 36 trials for the correlation calculation.

The total number of trials used for the correlation calculations is different from participant to participant. A participant sometimes touched the top plate of the apparatus after detachment, which negatively affects force data collection. In such cases, we asked the participant to conduct additional trials to make up for this error. Furthermore, we excluded all trials that had problems in data recording.

##### 2.4.4.1. Subject removal

Finally, we could not include any of the data collected from one female participant. In most of this individual's trials, the measured z-force did not smoothly converge to zero after detachment. Instead the signal persistently oscillated at a frequency that we suspect to be the resonance frequency of the force sensor and contact platform. We believe too much x-force (median *F*_x,rms_ = 1.48 N) was applied to the force sensor in an extremely short time (median *t*_pull_ = 0.022 s). These oscillations hindered the calculation of the parameters used to derive the physical variables for this subject, leaving a total of nine participants (two women, seven men).

## 3. Results

This section presents the results of our study, starting with the distributions of the measured physical variables and the reported stickiness ratings; the full data set can be viewed in the Supplementary Materials. The section then moves to correlation analyses both within and across participants, and it concludes with a detailed investigation of the effects of frictional forces in the studied interaction.

### 3.1. Physical Variables

**Figure 5:**
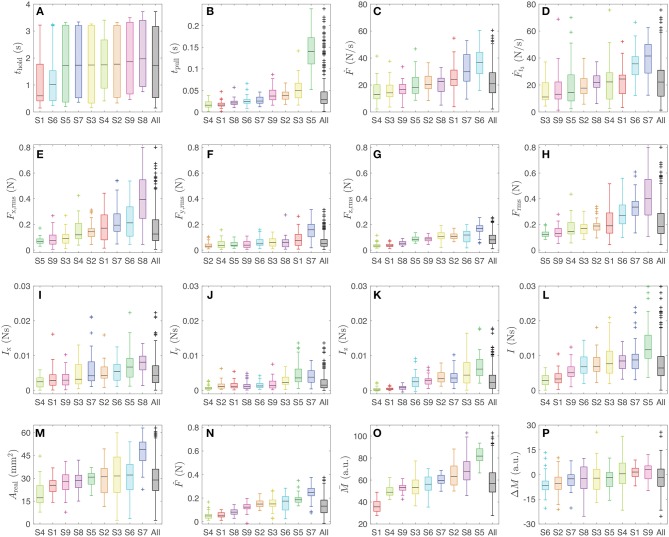
Boxplots of the sixteen physical variables calculated for each participant (S1–S9), ordered by ascending median value with all subjects combined at the end. The data are color-coded by participant. In each distribution, the black line shows the median, the box shows the second and third quartiles, the whiskers show the range up to 1.5 times the interquartile range, and the + symbol indicates outliers. The plotted variables are **(A)** *t*_hold_, **(B)** *t*_pull_, **(C)** Ḟ, **(D)** Ḟ_*t*_3__, **(E)** *F*_x,rms_, **(F)** *F*_y,rms_, **(G)** *F*_z,rms_, **(H)** *F*_rms_, **(I)** *I*_x_, **(J)** *I*_y_, **(K)** *I*_z_, **(L)** *I*, **(M)** *A*_real_, **(N)** $\hat{F}$, **(O)** $\bar{M}$, and **(P)** Δ*M*.

Figure 5 presents the distributions of the sixteen physical variables calculated from the data of all nine participants. In the case of *t*_hold_ (Figure 5A), the measured ranges are similar among participants because the signal to detach the finger appeared a random duration of either 0, 1.5, or 3 s after the participant reached a pressing force of 1.5 N. However, the ranges of all the other variables vary more because there were no external restrictions on these other aspects of the finger interaction.

### 3.2. Stickiness Perception Ratings

**Figure 6:**
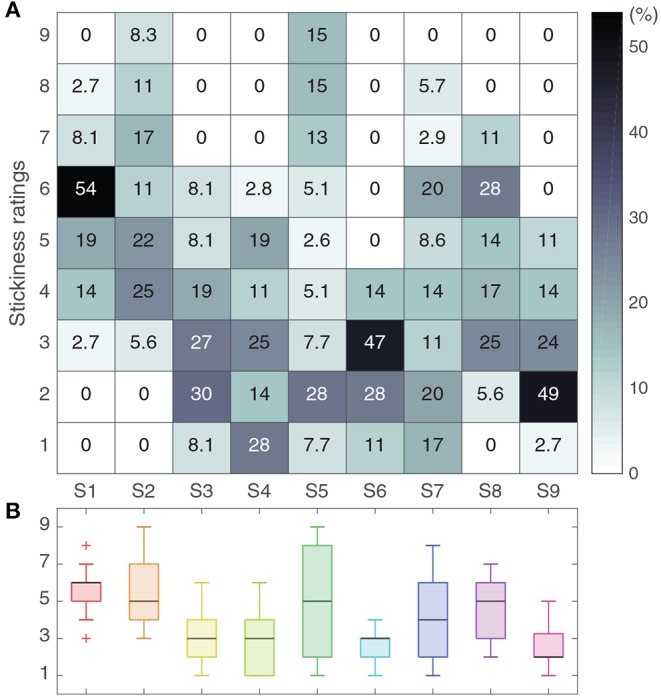
Distributions of stickiness ratings across participants. **(A)** The numbers in the heatmap represent the percentage of trials that received each rating. **(B)** The entire distribution of ratings per participant is shown as a boxplot.

Participants also had different rating distributions. As shown in Figure 6, one participant (S5) used the whole rating range, whereas another participant (S6) rated stickiness within a much more limited range (4 levels out of 9). The minimum and maximum ratings are also different between participants. For example, the rating range of S1 is from 3 to 8, but that of S3 is from 1 to 6. As we did not restrict any physical conditions except for the randomly allocated *t*_hold_, it may have been difficult for participants to determine whether a given trial deserved the overall maximum (or minimum) stickiness rating.

### 3.3. Correlation to Stickiness Within Participants

**Figure 7:**
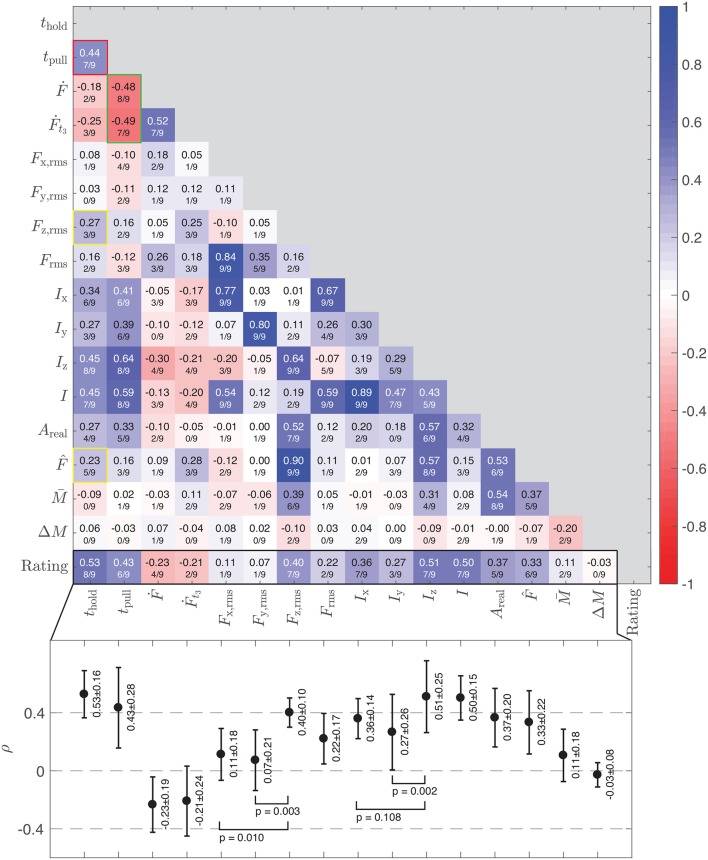
A heatmap showing the mean participant-specific correlation coefficients ($\bar{\rho }$) among the physical variables and the stickiness ratings. The second line of text in each cell lists the proportion of subjects whose correlations showed high significance (*p* < 0.05). The bar chart located below the heatmap shows the means and standard deviations of ρ across participants, plus the *p*-values of the four paired *t*-tests reported in the text.

The correlations between the 16 physical variable values and the stickiness ratings for each participant were calculated. One hundred and thirty six correlation coefficients (ρ) and corresponding probability values (p) were obtained per participant; all nine of these heatmaps can be viewed in the Supplementary Materials. Figure 7 shows a heatmap representing the average of the nine participant-specific heatmaps, where the ratio shown at the bottom of each box indicates the proportion of participants for whom the given correlation was significant (*p* < 0.05).

The bottom row in the heatmap (Figure 7) indicates how strongly each physical variable correlates with the stickiness rating. The means and standard deviations of the nine subject-specific correlation coefficients are plotted below the heatmap. Here, the pre-detachment pressing time (*t*_hold_, $\bar{\rho }=0.53$), the overall impulse during the normal direction during the finger detachment after normal force changes sign (*I*_z_, $\bar{\rho }=0.51$), the overall impulse during the same time span (*I*, $\bar{\rho }=0.50$), the time taken for the finger to detach (*t*_pull_, $\bar{\rho }=0.43$), and the RMS of z-force (*F*_z,rms_, $\bar{\rho }=0.40$) are the main contributors to the perception of stickiness.

Considering the calculational similarity between *I* and *I*_z_ and the fact that *I*_z_ is more strongly correlated with stickiness than *I* is, we believe that the z-component of impulse may mainly influence the stickiness rating. Therefore, we seek to understand the two variables based on *I*_z_.

The correlation of z-impulse (*I*_z_) to the stickiness rating was higher than that of the RMS of z-force (*F*_z,rms_) or that of the peak pull-off force ($\hat{F}$). This result indicates that the time taken for a finger to detach helps a person perceive stickiness; the fact that *t*_pull_ has a higher correlation coefficient to the stickiness rating than *F*_z,rms_ supports the importance of time involvement to evoke the feeling of stickiness.

### 3.4. Correlation Across Participants

**Figure 8:**
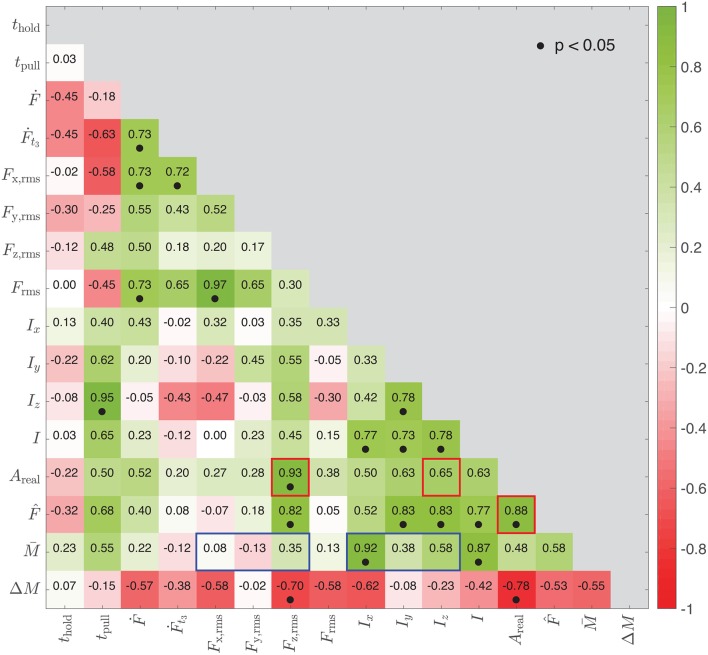
Correlations between the physical variables across participants based on the median variable value for each participant. A high value means that the nine participants are ranked in approximately the same order in the two corresponding subplots of Figure 5. A dot appears under the coefficient when the correlation is significant (*p* < 0.05).

We used the values of the sixteen physical variables to generate another heatmap that shows correlations between the median values of the variables across participants. The number in each cell of Figure 8 indicates how close the participant order sorted in ascending median of one variable (visible in Figure 5) is to that of another variable. These correlation coefficients are also calculated by Spearman's rank-order correlation method.

### 3.5. Effects of Frictional Forces on the Stickiness Ratings

The present study was performed in active touch conditions and did not prevent the participant from moving the finger laterally while pulling off. The lateral (frictional) components of the pull-off action therefore resulted in non-negligible forces generated in the x- and y-directions. Such forces primarily occurred in the x-direction, with larger measured forces along this axis compared to the y- and z-directions (Figures 5E–G); the median RMS forces in the x-, y-, and z-directions were 0.1250, 0.0454, and 0.0800 N, respectively. This trend also appeared in the directional impulse values (Figures 5I–K); the median impulse values were 0.0043, 0.0015, and 0.0022 Ns in the x-, y-, and z-directions, respectively.

However, we found that the vertical (normal) component of force and impulse evokes the feeling of stickiness more than the horizontal (frictional) component. To evaluate the perceptual value of stickiness cues generated in the vertical (z) direction, we tested how the RMS of the z-force and the z-impulse correlated with the perception of stickiness. For the RMS of the force, we found that *F*_z,rms_ correlated significantly better with the stickiness ratings than *F*_x,rms_ (paired *t*-test: *t* = 3.358, *df* = 8, *p* = 0.010) and *F*_y,rms_ (paired *t*-test: *t* = 4.292, *df* = 8, *p* = 0.003; see the bottom plot in Figure 7). For the impulse, *I*_z_ correlated significantly better with the stickiness perception than *I*_y_ (paired *t*-test: *t* = 4.345, *df* = 8, *p* = 0.002), but the difference with *I*_x_ did not reach statistical significance (paired *t*-test: *t* = 1.805, *df* = 8, *p* = 0.108).

**Figure 9:**
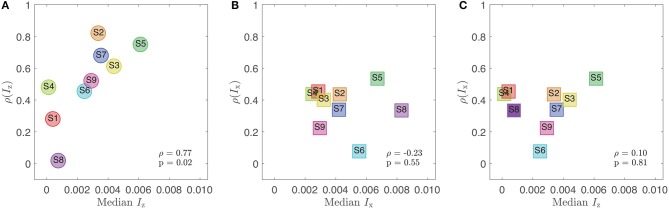
Sensory relevance between *I*_z_ and *I*_x_ by comparing correlations between stickiness and the impulse that participants felt. **(A)** A clear sensory relevance is shown by a high correlation between the directional correlation between stickiness and the z-impulse and the median z-impulse value experienced by participants. **(B)** There is no trend in the same correlation for the x-direction. **(C)** The correlation between stickiness and the x-impulse also does not correlate with the median x-impulse.

We then tested which directional component provides a better correlation between stickiness ratings and the median impulse of the measurements (i.e., the median x- or z-impulse from the measurements of each participant). We found that z-impulse's correlation becomes stronger as the median z-impulse increases (Spearman's correlation: ρ = 0.77, *p* = 0.02; see Figure 9A). On the contrary, the x-component did not show any sensory relevance (Spearman's correlation: ρ = −0.23, *p* = 0.55; Figure 9B). The correlation of *I*_x_ to stickiness also did not increase in terms of the participant's median *I*_z_ (Figure 9C). These results suggest that the sensory cues used by participants to shape their perception were mostly related to the vertical (normal) component of detachment and not very influenced by simultaneously generated frictional cues.

## 4. Discussion

As anticipated, subjects assigned a wide range of stickiness ratings to their interactions with the glass plate. The mechanics of the interaction also varied significantly, allowing us to uncover the underlying mechanical sources of this perceptual variability.

### 4.1. Main Perception Mechanism

We found that the perceived stickiness of a glass plate is significantly affected by the values of *t*_hold_, *t*_pull_, and *I*_z_. Simple logic and pieces of evidence found from the correlation heatmap (Figure 7) indicate that the main flow of the variables is *t*_hold_ → *I*_z_(*t*_pull_) → Rating. The contact time (*t*_hold_) is the variable that builds the adhesion between the fingerpad and the glass plate. This adhesion requires either a high normal force to pull off the glass or a high normal impulse; *t*_pull_ correlates more strongly with *t*_hold_ [$\bar{\rho }\left(t_{\text{hold}},t_{\text{pull}}\right)=0.44$, see red-bordered box in Figure 7] than the z-forces do [$\bar{\rho }\left(t_{\text{hold}},F_{\text{z},\text{rms}}\right)=0.27$, $\bar{\rho }\left(t_{\text{hold}},\hat{F}\right)=0.23$, see yellow-bordered boxes in Figure 7]. As *t*_pull_ is a component of *I*_z_, the high correlation coefficient implies that *t*_hold_ contributes more to the creation of the z-impulse than the z-forces do. Therefore, we conclude that *t*_hold_ affects *t*_pull_ by establishing secure contact, which causes an increase in the z-impulse (*I*_z_) and evokes a feeling of stickiness.

We believe that the time taken to break contact with the glass (*t*_pull_) is important for participants to feel stickiness because the time-related variables (*t*_pull_ and *I*_z_) are more strongly correlated with the stickiness ratings than the force-based variables are. According to Figure 7, detachment rate anti-correlates with pull-off time *t*_pull_ [$\bar{\rho }\left(Ḟ,t_{\text{pull}}\right)=-0.48$, $\bar{\rho }\left({Ḟ}_{t_{3}},t_{\text{pull}}\right)=-0.49$; see the boxes with green borders in Figure 7]. In the case of viscoelastic material, such as the fingerpad, the strategy of decreasing detachment speed contradicts the theoretical prediction for how to increase the force needed to separate two contacting objects (i.e., $\hat{F}$) (Barquins et al., 1978). However, given that the perception of stickiness is more related to *I*_z_ than to $\hat{F}$, it seems people are sensitive to the finger's separation time when judging stickiness. Thus, slow detachment increases the sticky feeling as long as the contact between the finger and the glass plate has been strongly established.

### 4.2. Finger Size and Peak Pull-Off Force

The difference in *A*_real_ across participants stems not only from contact conditions but also from the size of the participant's finger (see Figure 5M). We note that the participant order based on the median *A*_real_ is highly similar to their order based on the median *F*_z,rms_ values (see Figures 5G,M); indeed, the order similarity between these two variables is 0.93 (see Figure 8). Interestingly, the participant order by median *A*_real_ also has a close correlation to that by the median peak pull-off force [$\rho \left(\hat{F},A_{\text{real}}\right)=0.88$] but a much lower correlation to that by median z-impulse [ρ(*I*_z_, *A*_real_) = 0.65, highlighted with red boxes in Figure 8]. This close relationship between contact area (*A*_real_) and pull-off force (*F*_z,rms_, $\hat{F}$) across subjects provides good support for contact-adhesion theories (Gay, 2002; Barthel, 2008; Pastewka and Robbins, 2014). Since these variables were not strongly correlated with stickiness rating within subjects, it seems that perception of stickiness diverges somewhat from theory. Combined, our findings show that perceptual stickiness is distinct from mechanical stickiness; people base their ratings mainly on the impulse that they feel during detachment, not on the peak pull-off force.

### 4.3. Finger Moisture and Impulse

In the case of participant order by median impulse, another important variable is finger moisture; correlations of mean moisture to impulse are generally higher than to forces (highlighted with blue boxes in Figure 8). The low correlation coefficients between moisture and forces are anticipated by Cornuault et al.'s previous study showing that the water descriptor index of human fingers is not significantly correlated with the coefficient of friction on sticky surfaces (Cornuault et al., 2015). The close correlation between the finger moisture and impulse seems to imply that an increase in moisture leads to longer detaching duration. However, this correlation did not appear in Figure 7, which focuses on within-participant variations. In other words, the correlation to impulse is more pronounced at when considering a broad range of moisture levels across participants, rather than with the smaller variation of moisture within a participant (Figure 5O). This comparison suggests that substantial increases in finger moisture lead to longer detaching durations and consequently larger impulses.

### 4.4. Comparisons With Other Perceptual Studies

Other researchers have reported that rating distributions for roughness are similar to those for stickiness in experiments where the participant slides his or her finger across varied surface textures (Bensmaïa and Hollins, 2005). During such finger sliding, the perception of stickiness increases mainly due to the coefficient of friction (Bergmann Tiest, 2010), which is induced by the surface roughness (Tomlinson et al., 2009) and/or by liquids on the finger (Tang et al., 2015b). However, we showed that perceived stickiness can also vary in vertically active movements of a finger on a fixed surface, without any significant lateral motion.

Mith et al. showed that the human sensation of tackiness (stickiness) of an elastomer is similar to the full distance experienced between the fixed elastomeric sample and a pulling indenter (Mith et al., 2008), which is higher when the two materials stay attached for a longer duration of time. Interestingly, we found that the sensation of light stickiness made between a human finger and a hard surface also greatly depends on the time taken for the finger to detach, as well as on the impulse, which is intimately linked to detachment time. Although the surfaces being contacted in these two studies are quite different, we are encouraged by the alignment of these perceptual and mechanical results.

### 4.5. Experimental Limitations

Our conclusions are certainly limited by the population of participants in this study. We tested only a limited number of individuals in early-to-mid adulthood (29 ± 6.4), so we cannot know whether the significant mechanical and perceptual results we found would also hold for much younger or older populations, nor for a more diverse sample of individuals from the same age group. Given the design of our study, we also were not able to make conclusions about stickiness perception at normal forces that are lower or higher than 1.5 N.

Second, the test material was limited to one type of smooth glass. Because subjects knew that they were touching the same piece of glass in every trial, they might tend to report a constant value of stickiness that did not depend on trial-to-trial variations. We tried to minimize this bias by having each subject touch an identical glass plate under diverse pressing conditions before data collection began. In future research, it would be interesting to understand how important physical variables change depending on the test material. This comparison would give us a more profound understanding of the perceptual mechanism of light stickiness.

Third, research on this topic would benefit from an objective metric that can evaluate the perception strength against a stimulus. Our study could not elucidate how two independent people perceive the same interaction because the perception ratings were different from participant to participant. One possible reason for these rating variations is that the fingerpad's physiological composition varies greatly across people [e.g., hydrolipid film composition (Cornuault et al., 2015)]. Another possible explanation is that individuals were free to decide how to interpret the nine-point rating scale. If the assessment of perception is based on objective indicators, such as the activation strength of a particular brain region (Kim et al., 2017), physical variables measured from many people can be more efficiently used to derive an objective understanding of perception.

We were also limited by current moisture-sensing technology. There are no available transparent commercial sensors that can measure finger moisture. Thus, we placed a reliable commercial moisture sensor next to the glass plate. This configuration cannot prevent the loss of moisture on the fingerpad while the finger moves away from and to the sensor. In addition, measuring only before and after each trial did not let us study the effects of changes in the moisture over time due to sweat secretion and occlusion. To simultaneously measure the real contact area and the moisture of the finger during contact with the glass plate, we must develop a transparent moisture sensor.

## 5. Conclusion

In this paper, we investigated the physical variables that affect the perception of light stickiness. A custom-made apparatus recorded 3-DoF forces over time, contact area over time, and the moisture level of the subject's fingerpad before and after each trial. The recorded data yielded sixteen physical variables, including RMS forces in the three directions, impulses in the three directions, and finger detachment rates. Based on the data from a total of 324 trials by nine participants and their corresponding stickiness ratings, we computed Spearman's correlation coefficients both within and across subjects. We found that the finger's pre-detachment pressing time (*t*_hold_), the time taken for the finger to detach (*t*_pull_), and the impulse in the pressing (normal) direction during the finger detachment after the normal force changes sign (*I*_z_) were significantly related to stickiness. We believe that a longer pressing duration caused a larger impulse during the detachment of the finger from the glass surface and thus a more vivid stickiness sensation. Overall, our results imply that perceptual stickiness may be different from mechanical stickiness.

## Data Availability Statement

All datasets generated for this study are included in the article/Supplementary Material.

## Ethics Statement

The Ethics Council of the Max Planck Society reviewed and approved this research study under HI protocol 18-05B. Written informed consent was obtained from all participants. All research data were collected and analyzed according to the approved experimental protocol.

## Author Contributions

SN developed the apparatus, carried out the experiments, collected and analyzed the data, created the figures, wrote the first draft of the manuscript, edited the manuscript, and implemented changes requested by the other authors. YV, DG, and KK helped to design the psychophysical experiment, gave input on data analysis, guided the creation of the figures, wrote short sections of text, and extensively edited the manuscript. Additionally, KK gave input on the apparatus design and supervised the project.

### Conflict of Interest

The authors declare that the research was conducted in the absence of any commercial or financial relationships that could be construed as a potential conflict of interest.

## Supplementary Material

Table S1-S9 Raw data collected from all subjects (S1–S9), including sixteen physical variables and one perception rating for each trial.
