# Supplementary material for: Physical Variables Underlying Tactile Stickiness During Fingerpad Detachment
Source: Front Neurosci. 2020 Apr 15;14:235. doi: 10.3389/fnins.2020.00235 (PMC7177046; doi:10.3389/fnins.2020.00235)
Supplement: Supplementary file 2 [file Data_Sheet_2.PDF]

Supplementary Material

CORRELATION HEATMAPS WITHIN PARTICIPANTS

We present the Spearman's correlation coefficients between the sixteen physical variable values and the stickiness ratings across trials for each participant. Including self-correlations and the correlations between the physical variables, a total of 136 correlation coefficients (ρ) and corresponding probability values (p) were obtained per participant. Every participant's figure shows a heatmap with cells that are labeled and colored by the correlation coefficients; the lower number in each cell is the associated p-value.

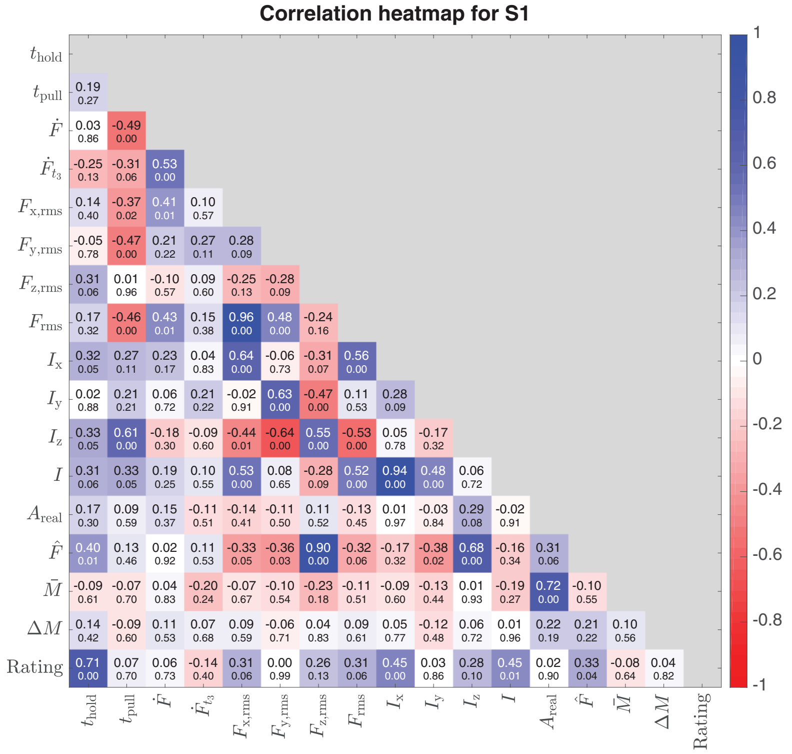

Figure S1. Correlation heatmap for S1.

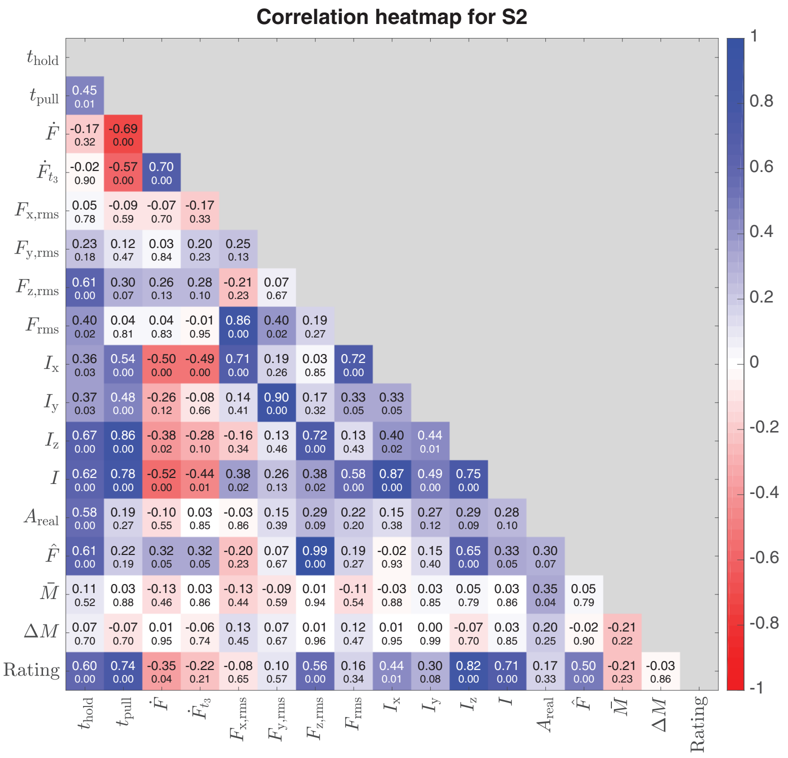

Figure S2. Correlation heatmap for S2.

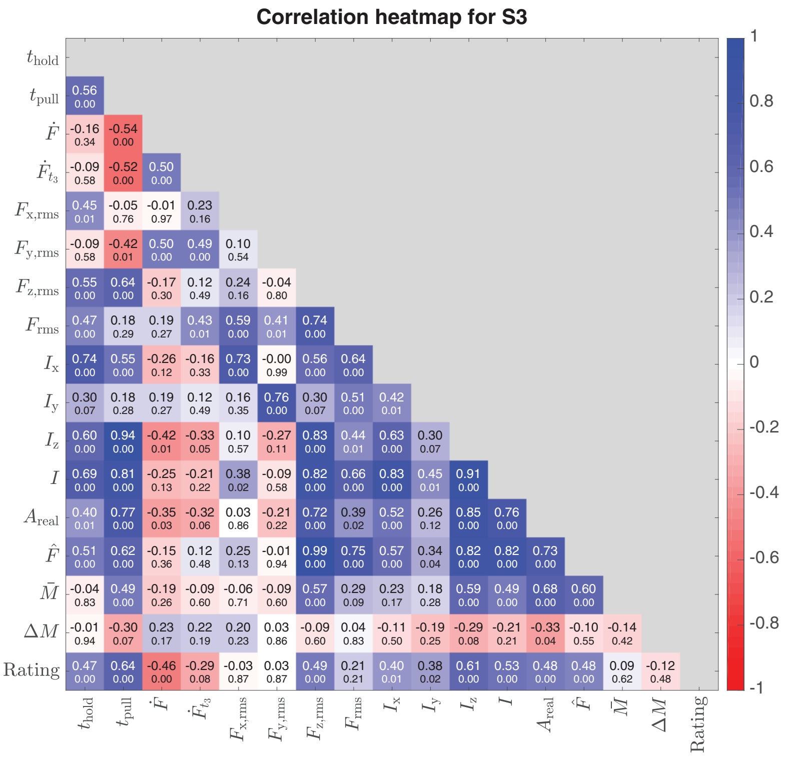

Figure S3. Correlation heatmap for S3.

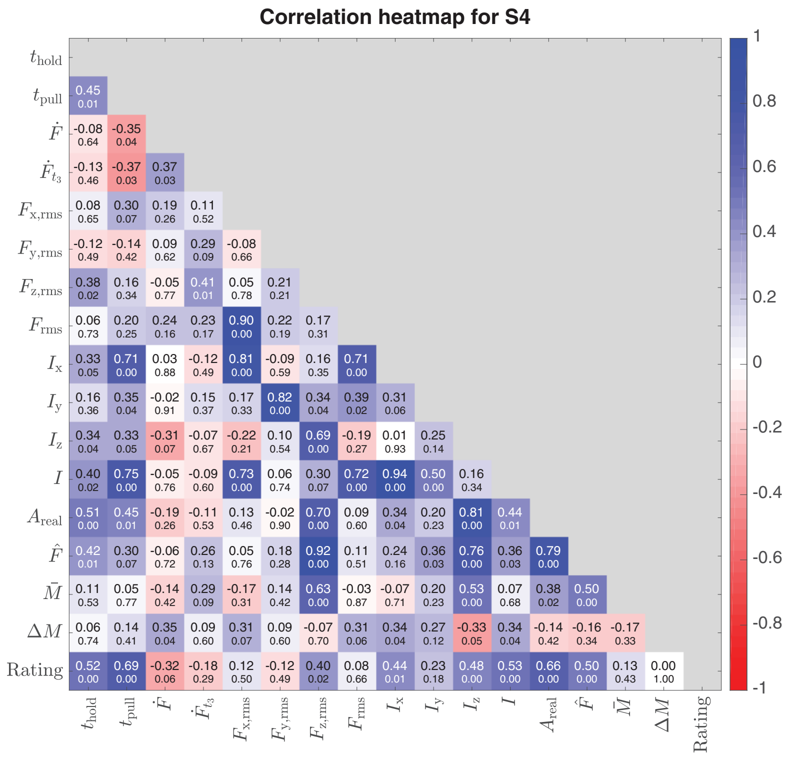

Figure S4. Correlation heatmap for S4.

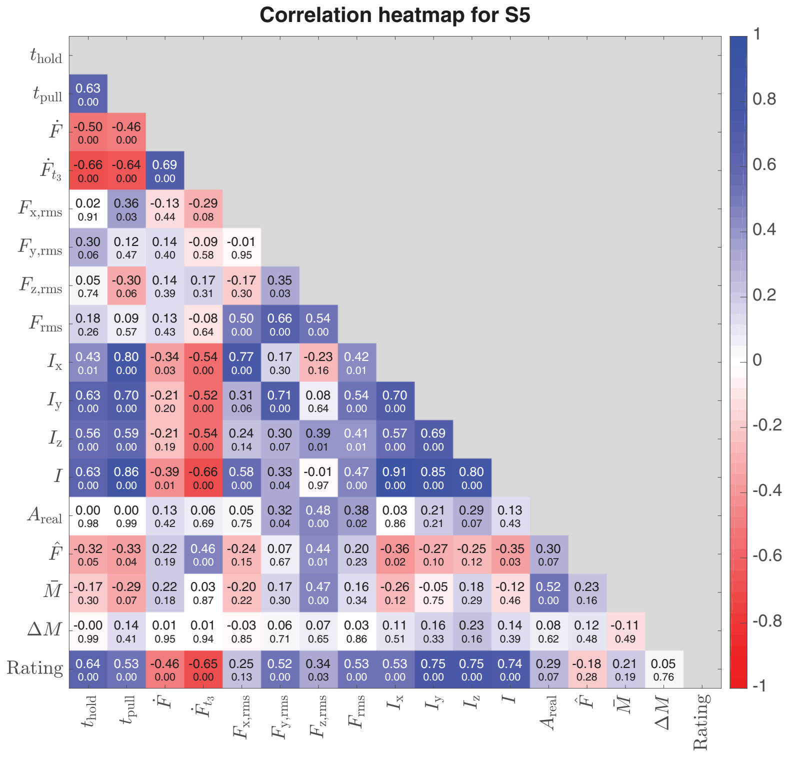

Figure S5. Correlation heatmap for S5

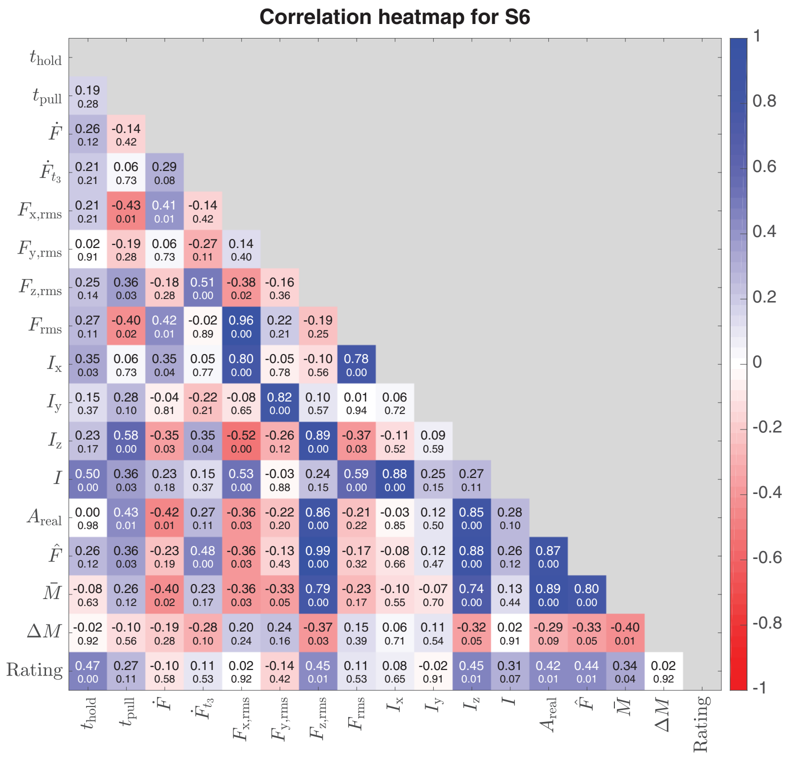

Figure S6. Correlation heatmap for S6

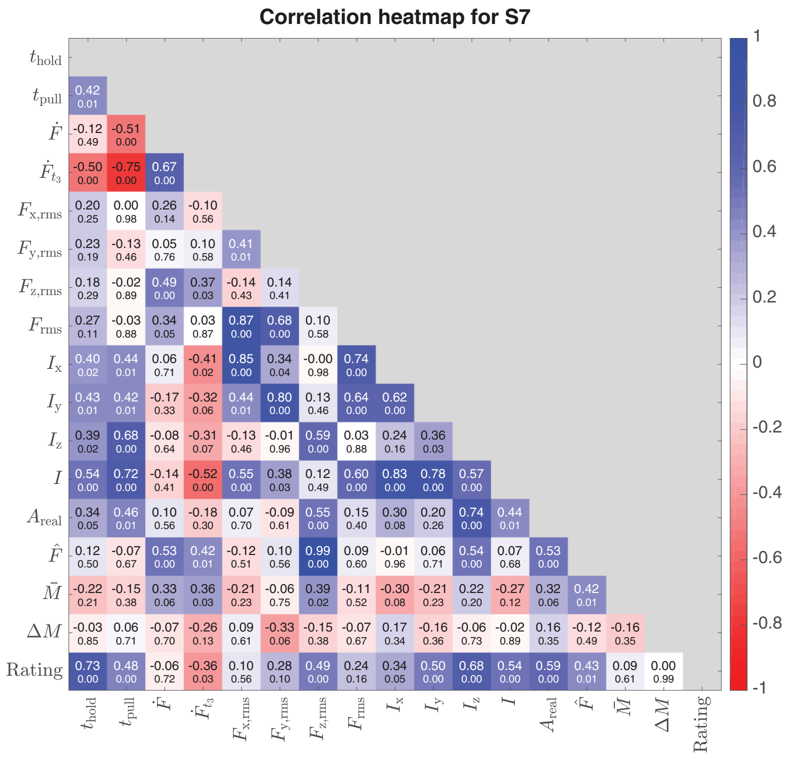

Figure S7. Correlation heatmap for S7

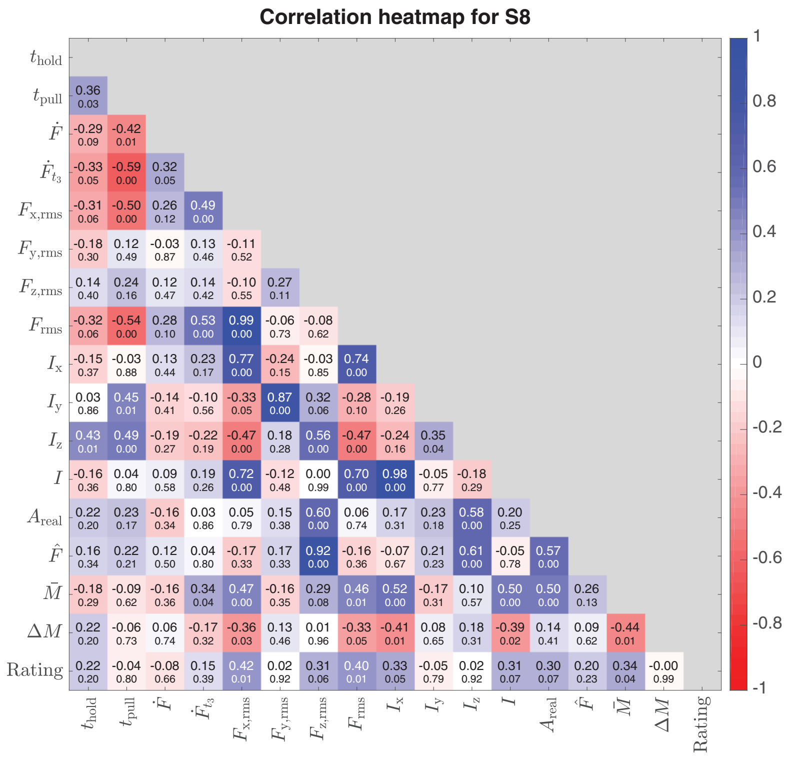

Figure S8. Correlation heatmap for S8

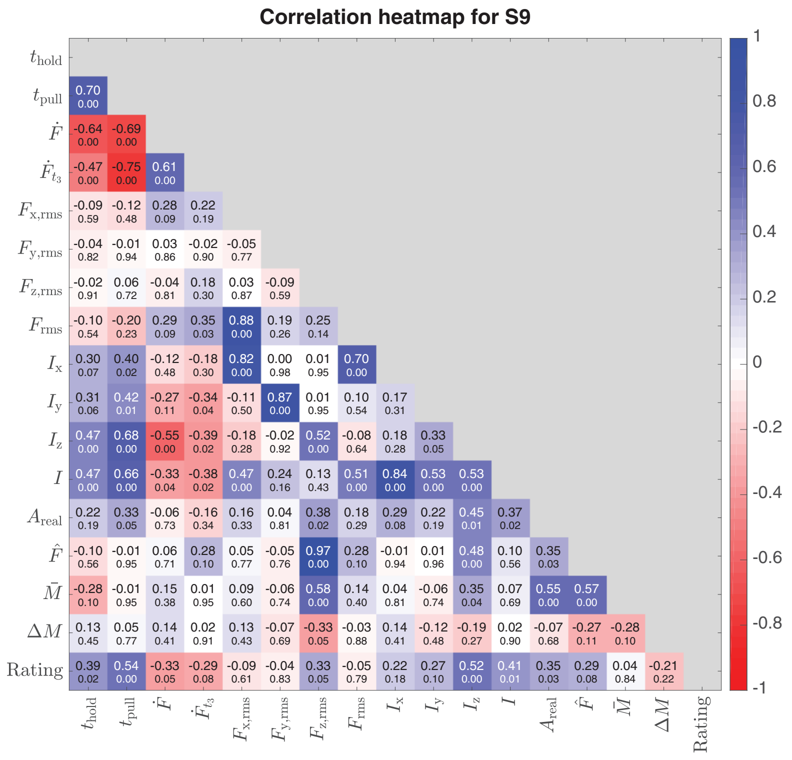

Figure S9. Correlation heatmap for S9
